# Short- and long-term follow-up results of daily 5-mg tadalafil as a treatment for erectile dysfunction and premature ejaculation

**DOI:** 10.1080/2090598X.2021.2024695

**Published:** 2022-01-23

**Authors:** Tarek Mohamed Gharib, Ibrahim Abdel-Al, Adel Elatreisy, Wael Kandeel, Waleed El-Shaer, Abdrabuh M Abdrabuh, Elsayed Mohamed Salih, Ahmed Sebaey

**Affiliations:** a Urology Department, Faculty of Medicine Benha University, Benha, Egypt; b Urology Department, Faculty of Medicine, Al-Azhar University, Assiut Branch, Egypt; c Urology Department, Faculty of medicine, Al-Azhar University, Cairo, Egypt

**Keywords:** Tadalafil, erectile dysfunction, premature ejaculation

## Abstract

**Objective:**

To evaluate the safety and effectiveness of daily 5-mg tadalafil treatment for men who have erectile dysfunction (ED) and premature ejaculation (PE), and to assess the long-term follow-up for ED and PE improvement persistence years after the cessation of medication.

**Patients and Methods:**

A prospective, single-blind, randomised study included 160 patients with ED and PE. All were evaluated using the International Index of Erectile Function (IIEF-5) questionnaire to evaluate ED and intravaginal ejaculatory latency time (IELT) for PE. Patients were subdivided into two equal groups. Group I (80 patients) treated with daily 5-mg tadalafil for 3 months, and Group II (80 patients) treated with a placebo for the same period. After 3 months of treatment and 2 years later after cessation of tadalafil, all patients were assessed for ED and PE.

**Results:**

The mean (SD) IELT and IIEF-5 score pre-treatment were 37 (11.24) s and 13.2 (4.2) for Group I, while in Group II they were 35.98 (10.8) s and 13.12 (4.11), respectively. After 3 months of treatment, the mean (SD) IELT in Group I showed a highly significant improvement from 37 (11.24) s to 120.5 (47.37) s (*P* < 0.001) but Group II showed no significant improvement from baseline to [39.43 (13.6) s; *P* > 0.05]. For the IIEF-5 score, there was a highly significant improvement from baseline to 20.45 (4.5) in Group I (*P* < 0.001), while there was no significant difference in Group II from baseline to [15 (4.84); *P* > 0.05]. At 2 years after cessation of tadalafil, there was statistically significant improvement in the IELT and IIEF-5 from baseline to endpoint .

**Conclusion:**

Oral daily 5-mg tadalafil was effective, tolerable, and safe treatment for patients with ED and PE. Long-term follow-up at 2 years confirmed the persistence of a significant improvement for both ED and PE.

**Abbreviations**: ED: erectile dysfunction; IIEF-5: five-item version of the International Index of Erectile Function questionnaire; IELT: intravaginal ejaculatory latency time; OAD: once-daily; PDE5i: phosphodiesterase-5 inhibitors; PE: premature ejaculation; PRN: *pro re nata*

## Introduction

Erectile dysfunction (ED) and premature ejaculation (PE) are the most common sexual dysfunctions with a prevalence of ~30% and ~20%, respectively [1,2]. ED is a failure to accomplish and maintain an adequate erection to reach satisfaction with sexual intercourse for the last 6 months, while PE is defined as an early ejaculation within ~1 min with minimal sexual excitation just after intravaginal penetration with involuntary control that has occurred for ≥6 months in all or almost all sexual activities, leading to anxiety and depression [3].

Sexual dysfunction includes ejaculatory and orgasmic disorders, ejaculatory disorders include PE and retarded ejaculation (RE), but orgasmic disorders include anorgasmia and hypo-orgasmia. PE is classified into primary or lifelong and secondary or acquired [3]. Organic factors are the commonest predictors for acquired PE, such as prostatitis [4] and endocrine disorders [5,6]. Still, routine hormonal testing should only be directed to the patient’s complaints and risk factors with specific findings from history or physical examination [7].

Every man with PE should be adequately screened for ED, and where present, this should be addressed first. Men with ED can have performance anxiety, which may also favour PE. Accordingly, treating men with ED with ED medications improves erections and ejaculatory latency times (ELTs) [7,8].

PE co-exists with ED in ~30% of patients, mainly secondary PE. However, the specific phenotype of ED-PE men has never been systematically investigated. The five-item version of the International Index of Erectile Function (IIEF-5) questionnaire and intravaginal ELT (IELT) is used to evaluate ED and PE, respectively. The IELT has higher sensitivity and specificity for the evaluation of PE [9].

There are many modalities for PE treatment, the most commonly used are behavioural and pharmacological therapy, but behavioural therapy is inefficient for many couples. Although many drugs are used for PE, serotonin reuptake inhibitors are the most common drugs used in PE. Other medications like topical anaesthesia or opioid agonists, like tramadol, are less commonly used [10].

Tadalafil with once-daily (OAD) and on-demand [*pro re nata* (PRN)] dose regimens is sufficient for treating ED. Other studies reported that the tadalafil OAD dose regimen is better, and more sexual satisfaction occurred than PRN [11,12]. Tadalafil, which is commonly used in the treatment of ED, has been recently investigated in some studies for treating PE, and most of these studies reported a significant improvement [13–15]. There were no data in the literature about long-term follow-up results of tadalafil on the improvement of PE.

The present study evaluated the efficacy of daily 5-mg tadalafil treatment vs placebo for 3 months on the erectile function (assessed using the IIEF-5) and ejaculation time (IELT) in patients with ED and PE. In addition, we investigated whether there was a significant improvement in the IIEF-5 and IELT after the stoppage of tadalafil for a long time.

## Patients and methods

A prospective, single-blind, randomised study comparing the safety and efficacy of 5-mg tadalafil continuous daily dosing for 3 months compared with placebo for ED with PE and assessment of persistence of improvement after 2 years from the cessation of tadalafil.

This study was conducted on 160 patients attending the urology outpatient clinic, Benha University Hospital, and Al-Azhar University Hospital from April 2018 to April 2019, including men aged 18–65 years who had ED and lifelong PE for the last 6 months of a continuous marriage relationship.

We excluded from our study patients with a neurogenic disorder, parkinsonism, diabetes mellitus, active Urinary tract infection (UTI), chronic prostatitis, chronic renal failure, Peyronie’s disease, and endocrine disorders, including low testosterone, hyperprolactinaemia, and thyroid dysfunction, patients taking medications for PE and drugs affecting erectile function.

Written informed consent was obtained from all patients enrolled in this study, which the local ethics committee approved.

The sample size was calculated using the EPI info 2000 statistical package (Centers for Disease Control and Prevention (CDC), Atlanta, GA, USA). The null hypothesis of the study was that tadalafil has no treatment effect relative to placebo. A statistical significance at *P* < 0.05 was required for the two co-primary endpoints (IIEF-5, IELF) to reject the null hypothesis, and a sample size of 160 patients (80 placebo and 80 tadalafil) was calculated to give 90% power to detect a significant treatment effect. Patients were subdivided into two equal groups and randomised using the secure envelope method. Group I included 80 patients treated with a daily 5-mg tadalafil oral tablet for 3 months, and Group II included 80 patients treated with a placebo for the same period. The tadalafil group were followed up for 2 years after cessation of tadalafil, but five patients were lost during follow-up, and so 75 patients were assessed at the end of 2 years.

Full medical and sexual history was taken with the history of current medications, a complete physical and genital examination was performed, and all patients were evaluated using the IIEF-5 to evaluate ED and IELT for PE [16] before and after treatment and at the end of the 2-year follow up.

The IIEF is a multidimensional validated questionnaire including 15 questions in the five domains of sexual function (erectile and orgasmic functions, sexual desire, satisfaction with intercourse, and overall sexual satisfaction). More recently, to simplify the IIEF, an abridged five-items version the IIEF-5 has been used as a diagnostic tool for ED and evaluation of treatment. It consists of only five questions, and each IIEF-5 item is scored on a 5-point ordinal scale where lower values represent an impaired sexual function. According to this scale, ED is classified into three grades based on IIEF-5 scores: severe ED (score: 1–7), moderate ED (8–11), and mild ED (12–21) [17].

The IELT was defined as the time from intravaginal intromission to intravaginal ejaculation. Female partners were provided with a stopwatch and instructions on how to record and measure the IELT. It can be used as an integrated measurement of the partners, and that it was a simple and objective screening indicator for diagnosing self-reporting PE [18].

### Statistical analysis

The collected data were revised, organised, tabulated, and statistically analysed using the Statistical Package for the Social Sciences (SPSS®) version 25.0 (IBM Corp., Armonk, NY, USA). Data are presented as the mean ± standard deviation (SD); Continuous variables in comparable groups were compared by the Student’s *t*-test (two-tailed). In the intention-to-treat (ITT) analysis, all randomised patients were included in their original group irrespective of their compliance with treatment or loss to follow-up. Patients who were lost to follow-up were considered as a failure to treat. The level of significance was accepted for *P* < 0.05.

## Results

Both groups were comparable for age, weight, and height (Table 1). At the end of 3 months, the mean (SD) IELT in Group I showed a highly significant improvement from 37 (11.24) to 120.5 (47.37) s (*P* < 0.001). While in Group II, the mean (SD) IELT showed no significant improvement from baseline at 35.98 (10.8) to endpoint at 39.43 (13.6) s (*P* > 0.05).

**Table 1.** Patient demographics and baseline and clinical characteristics: tadalafil vs placebo (*N* = 160)

Characteristic	Tadalafil *N* = 80	Placebo *N* = 80	P*
Age, years, mean (SD)	36.3 (11.6)	37.1 (12)	0.68
Range (max – min)	41(18–59)	40(18–58)	
Weight, kg, mean (SD)	78.9 (8.4)	80.3 (10.3)	0.52
Range (max – min)	19 (69–88)	18 (71–89)	
Height, cm, mean (SD)	171.3 (14.6)	174 (12.5)	0.77
Range (max – min)	22(159–181)	21(160–181)	
IELT at baseline, s, mean (SD)	37 (11.24)	35.98 (10.8)	0.55
Range (max – min)	47(10–57)	48(10–58)	
IIEF-5 score at baseline, mean (SD)	13.2 (4.2)	13.12 (4.11)	0.94
Range (max – min)	16 (4–20)	16 (4–22)	

*Independent sample *t*-test.

For the IIEF-5, there was a highly significant improvement from a mean (SD) IIEF-5 score of 13.2 (4.2) at baseline to 20.45 (4.5) at endpoint in Group I (*P* < 0.001), while there was no statistically significant difference in Group II from baseline at 13.12 (4.11) to 15 (4.84) at endpoint (*P* > 0.05; Table 2).

**Table 2.** Comparison of patients’ clinical characteristics at baseline and endpoint: tadalafil vs placebo (*N* = 160)

Parameters	Tadalafil *N* = 80			Placebo *N* = 80			++*P*2
	Baseline	Endpoint	Change	Baseline	Endpoint	Change	
IELT, s, mean (SD)	37 (11.24)	120.5 (47.37)	–83.47	35.98 (10.8)	39.43 (13.6)	–3.45	<0.001
+*P*1	*<0.001 HS*			*0.042 S*			
IIEF-5 score mean (SD)	13.2 (4.2)	20.45 (4.5)	–7.27	13.12 (4.11)	15 (4.84)	–1.87	<0.001
+*P*1	<0.001			0.013			

*P* value is significant if *P* < 0.05.

+*P*1: Baseline vs endpoint, paired sample *t*-test.

++*P*2: Endpoint of tadalafil vs placebo group, independent sample *t*-test.

For the IIEF-5 scores, the patients with ED were divided into three grades as severe ED (score: 1–7), moderate ED (8–11), and mild ED (12–21). All grades of ED showed a statistically significant difference in IELT in Group I (*P* < 0.01) vs placebo (Group II). However, there was no significant difference between the grades of ED from baseline to endpoint (*P* = 0. 0.29 and *P* = 0.46; Tables 3 and 4).

**Table 3.** Comparison of ED groups in terms of IELT before and after daily 5-mg tadalafil treatment (*N* = 80)

IELT, s, mean (SD)	Tadalafil *N* = 80			+*P*1
	Baseline	Endpoint	Change	
Mild ED, *n* = 47	37.5 (12.2)	127.85 (46.7)	90.35	<0.001
Moderate ED, *n* = 26	34.8 (10.1)	121.5 (45.7)	86.7	<0.001
Severe ED, *n* = 7	42.14 (6.9)	67.6 (18.98)	25.46	<0.001
++*P*2	0.29	0.46		

*P* value is significant if *P* < 0.05.

+*P*1: Baseline vs endpoint, paired sample *t*-test.

++*P*2: Comparison of IELT 3 scores, F-test (ANOVA).

**Table 4.** Comparison of ED groups in terms of IELT before and after placebo (*N* = 80)

IELT, s, mean (SD)	Placebo *N* = 80			+*P*1
	Baseline	Endpoint	Change	
Mild ED, *n* = 49	36.24 (11.8)	37.6 (13.8)	1.36	>0.05
Moderate ED, *n* = 24	33.7 (8.9)	42.5 (13.8)	8.8	0.005
Severe ED, *n* = 7	42.14 (6.86)	41.85 (11.13)	0.29	>0.05
++*P*2	0.18	0.15		

*P* value is significant if *P* < 0.05.

+*P*1: Baseline vs endpoint, paired sample *t*-test.

++*P*2: Comparison of IELT 3 scores, F-test (ANOVA).

At the end of the 2-year follow-up, after the cessation of tadalafil, there was a significant improvement in IELT and IIEF-5 form baseline [mean (SD) 37 (11.24) s and 13.2 (4.2), respectively] to endpoint [mean (SD) 98 (18.3) s and 19.1 (2.3), respectively]. However, the IIEF-5 and IELT values decreased slightly compared with the previous values after 3 months of treatment (Table 5).

**Table 5.** Comparison of patient clinical characteristics at baseline and after 2 years of 5-mg tadalafil administration (*N* = 75)

Variable	Tadalafil *N* = 75			*P*
	Baseline	Endpoint	Change	
IELT, s, mean (SD)	37 (11.24)	98 (18.3)	–61	<0.001
IIEF-5 score, mean (SD)	13.2 (4.2)	19.1 (2.3)	–5.9	<0.001

*P* value is significant if *P* < 0.05, independent sample *t*-test.

The common side-effects were headache in 13 patients (16.25%), lower back pain in 11 (13.75%), dyspepsia in eight (10%), gastro-oesophageal reflux in four (5.0%), and myalgia in three (3.3%). Most of the side-effects disappeared spontaneously over time.

## Discussion

The relationship between ED and PE was hypothesised by Jannini et al. [19], as he declared a continuous circle between both PE and ED as the trial to delay ejaculation decreases stimulation and produces ED. The attempt to attain an erection by constant repeated stimulation ends in rapid ejaculation and stress with frustration. In this scenario, PE occurs secondary to increased sexual arousal to attain erection with anxiety that increases sympathetic stimulation and produces PE [19].

Our present study investigated the effect of daily 5-mg tadalafil on ED and PE after 3 months of medical treatment. It showed a significant improvement in erectile function and overall sexual satisfaction with significant improvement in IIEF-5 scores and IELT compared to placebo. In addition, all ED grades (mild, moderate, and severe) showed a statistically significant improvement in IELT from before to after treatment. However, it was not statistically significant between the grades.

The first study to assess the effect of 5-mg tadalafil daily on PE was done by Ozcan et al. [20]. They found a significant improvement in IELT in patients with lifelong PE. These results comply with our present results that showed improved in IELT up to 2.3 min.

In a retrospective study, Karabakan et al. [21] found that 5-mg tadalafil alone for 3 months significantly improved all measured parameters (all *P* < 0.001). The study included 60 patients diagnosed with ED with mean (SD) baseline scores of 2.2 (1.4) min for IELT and 9.5 (3.7) for the IIEF-5 and at the endpoint was 3.4 (1.9) min and 16.1 (4.7) for IELT and the IIEF-5, respectively. Also, tadalafil increased the IELT in all three ED groups (severe, moderate, and mild ED groups). In addition, there was a statistically significant difference between the pre- and post-treatment values of IELT variables (*P* < 0.01) in all groups. Still, there was no statistically significant difference between the ED groups in terms of IELT, and these results were comparable with our study. Also, our present results agree with Salonia et al. [22] who reported a significant improvement in IELT and overall sexual satisfaction and decreased stress.

Our present findings disagree with the results of McMahon et al. [23] who performed a well-designed study to assess the efficacy of sildenafil in men with PE compared to placebo; they found increased overall sexual satisfaction with improved control over ejaculation but without a significant improvement in IELT.

Another double-blind placebo-controlled study done by Mattos et al. [24] to assess the effect of 20-mg tadalafil alone or with fluoxetine in PE revealed a better improvement in combined tadalafil and fluoxetine than each drug alone.

In our present study, most of the side-effects were mild and tolerable. No patient discontinued the medications due to these side-effects in the form of headache, myalgia, and lower back pain, which agrees with Karabakan et al. [21] who reported mild symptoms that disappeared over time, including headache, muscle, low back pain, and flushing.

To our knowledge, the present study is the first to assess the long-term effect of daily 5-mg tadalafil on ED and PE after 2 years from medication cessation and revealed persistence of significant long-term improvement that proves the presence of a continuous circle and co-existence of ED and PE.

The limitation of our study is that it is a single-blind study with a small sample size. A double-blinded randomised study based on subjective and objective evaluation tools for sexual functional outcomes and a larger population is recommended.

We conclude that a daily 5-mg tadalafil oral tablet is an effective, tolerable, and safe treatment option for patients with ED and PE. In addition, we noticed significant long-term improvement after the cessation of tadalafil.

## Main points

1. PE co-exists with ED in 30% of patients.
2. The IIEF-5 and IELT can be used to evaluate ED and PE, respectively.
3. IELT has higher sensitivity and specificity for the evaluation of PE.
4. Daily 5-mg tadalafil for 3 months is an effective, tolerable, and safe treatment for patients with ED and PE, which is also still effective 2 years after cessation. So we advise, from the results of our present study, implementing daily 5-mg tadalafil for 3 months in patients with both ED and PE.
